# DECIMER-Segmentation: Automated extraction of chemical structure depictions from scientific literature

**DOI:** 10.1186/s13321-021-00496-1

**Published:** 2021-03-08

**Authors:** Kohulan Rajan, Henning Otto Brinkhaus, Maria Sorokina, Achim Zielesny, Christoph Steinbeck

**Affiliations:** 1 Institute for Inorganic and Analytical Chemistry, Friedrich-Schiller-University Jena, Lessingstr. 8, 07743 Jena, Germany; 2 Institute for Bioinformatics and Chemoinformatics, Westphalian University of Applied Sciences, August-Schmidt-Ring 10, 45665 Recklinghausen, Germany

**Keywords:** Deep learning, Image Segmentation, Optical Chemical Structure Recognition, Neural Networks, Chemical data extraction

## Abstract

Chemistry looks back at many decades of publications on chemical compounds, their structures and properties, in scientific articles. Liberating this knowledge (semi-)automatically and making it available to the world in open-access databases is a current challenge. Apart from mining textual information, Optical Chemical Structure Recognition (OCSR), the translation of an image of a chemical structure into a machine-readable representation, is part of this workflow. As the OCSR process requires an image containing a chemical structure, there is a need for a publicly available tool that automatically recognizes and segments chemical structure depictions from scientific publications. This is especially important for older documents which are only available as scanned pages. Here, we present *DECIMER (Deep lEarning for Chemical IMagE Recognition) Segmentation*, the first open-source, deep learning-based tool for automated recognition and segmentation of chemical structures from the scientific literature. The workflow is divided into two main stages. During the detection step, a deep learning model recognizes chemical structure depictions and creates masks which define their positions on the input page. Subsequently, potentially incomplete masks are expanded in a post-processing workflow. The performance of DECIMER Segmentation has been manually evaluated on three sets of publications from different publishers. The approach operates on bitmap images of journal pages to be applicable also to older articles before the introduction of vector images in PDFs. By making the source code and the trained model publicly available, we hope to contribute to the development of comprehensive chemical data extraction workflows. In order to facilitate access to DECIMER Segmentation, we also developed a web application. The web application, available at https://decimer.ai, lets the user upload a pdf file and retrieve the segmented structure depictions.
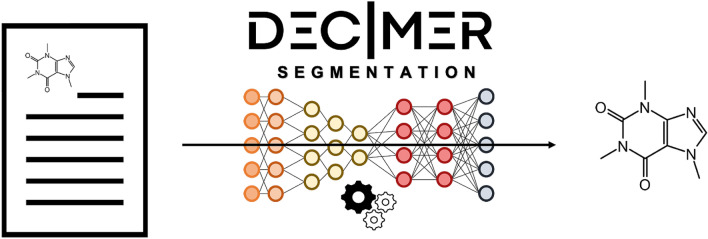

## Introduction

Chemical information is communicated as text and images in scientific publications [1]. These data formats are not intrinsically machine-readable and the manual extraction of chemical information from the literature is a time-consuming and error-prone procedure [2]. Hence, the increasing amount of chemical information being published creates a demand for automated chemical information extraction methods [3].

Over the course of the last three decades, there has been an active development in the field of Optical Chemical Structure Recognition (OCSR). OCSR is the translation of an image of a chemical structure into a machine-readable representation [4]. Most OCSR tools are only capable of processing images with pure chemical structure depictions. Consequently, an automated segmentation of chemical structures from surrounding document information (text, tables etc.) is desirable. Previous approaches for this task are briefly described in the following paragraphs.

The open-source OCSR tool OSRA was published with a rule-based page segmentation algorithm. This mechanism identifies a chemical structure depiction based on the dimensions of a rectangular bounding box around a region of interest and the ratio of black and white pixels within the bounding box [5].

The open-source tool ChemSchematicResolver (CSR) is capable of segmenting images which only contain labels and chemical structure depictions. The classification of objects as labels or structure depictions is done using k-means clustering based on a custom feature density metric. If the publication is available as a markup document, these images can be extracted automatically, so that CSR is capable of processing whole documents [6]. Nevertheless, CSR is incapable of handling scanned pages or images which contain other objects than labels and structure depictions.

In 2019, Staker et al. reported a deep-learning-based OCSR tool which contains a segmentation procedure [7]. Opposed to the previously mentioned feature density-based approaches used by OSRA and CSR, they trained a convolutional neural network based on the U-Net architecture [8] to address the segmentation problem. Every image is processed multiple times at different resolutions and the masks generated by the model are averaged. The model was trained on a semi-synthetic dataset: OSRA was used to identify bounding boxes of potential chemical structure depictions in an unspecified amount of publications and patents. These areas were then cut out of the original documents and replaced with structures from publicly available datasets. During training, the images were randomly modified (with e.g. binarization, brightness adjustments) for data augmentation purposes. The segmentation accuracy has not been reported independently and the accuracy for the whole process of segmentation and structure resolution on different training datasets has been reported to be between 41 and 83% [7]. Unfortunately, the authors have not made their code and the trained models openly available.

With the DECIMER [9] project, we are currently working on the development of an open-source platform for the automated chemical structure extraction from printed literature. It aims at segmenting all chemical structure depictions from a given scanned document from the printed scientific literature and resolving their identity to yield a machine-readable presentation of the molecule. Here, we present *DECIMER Segmentation,* the first step of the DECIMER project and the first openly available deep learning tool for the segmentation of chemical structure depictions from scanned whole-page documents. Perspectively, the segmented chemical structure depictions will be used as input for the DECIMER algorithm, an OCSR method which predicts the SMILES string of the depicted chemical structure.

**Fig. 1:**
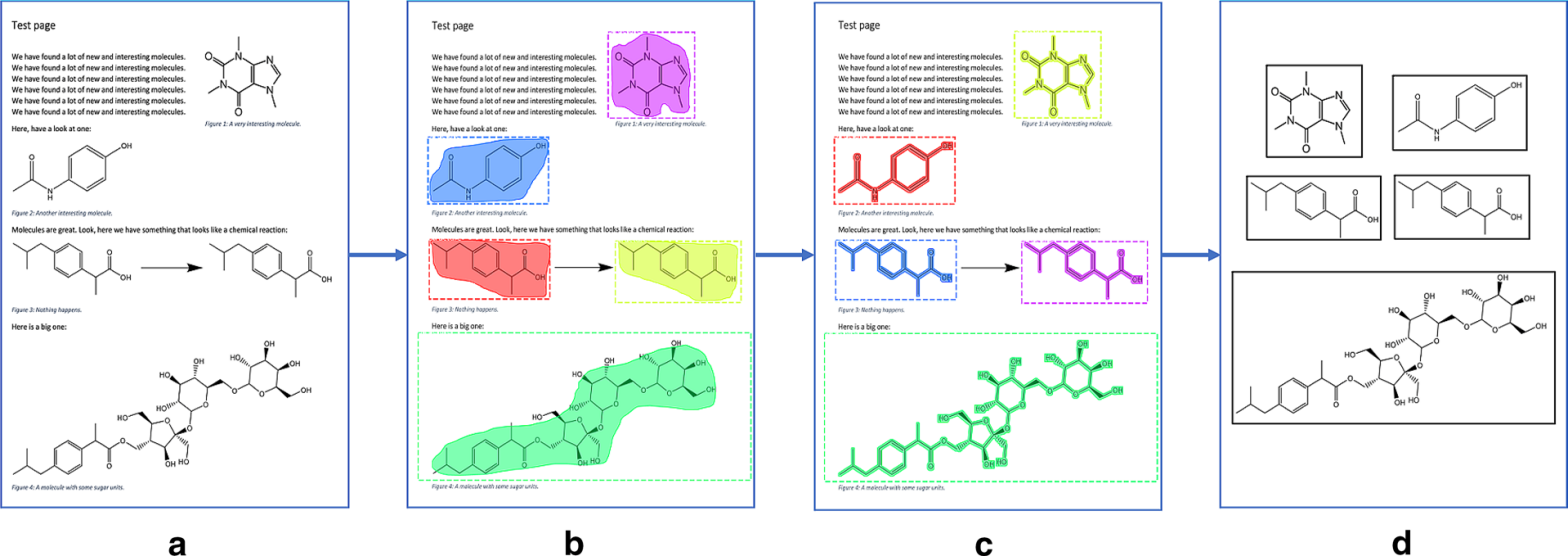
Graphical summary of the DECIMER Segmentation workflow. The input is an image of a page with chemical structure depictions (**a**). Then, the chemical structure depictions are detected using the Mask-RCNN model (**b**). Subsequently, the masks that define the positions of the depictions are refined and expanded (**c**). Finally, the regions defined by the masks are segmented to yield individual images of chemical structure depictions (**d**)

The algorithm consists of two main stages: First, during the detection step, a deep learning-based model generates masks that define the positions of the chemical structures in a given image. This is followed by a mask expansion procedure during which potentially incomplete masks are expanded until they cover the depictions completely (Fig. 1).

We did not attempt to extract vector graphics from modern PDF articles since this approach would fail for older articles before the early 1990′s, which are mostly scanned pages from printed versions of the journal. Instead, our approach operates on bitmap images of journal pages to be widely applicable also to older articles before the introduction of vector images in PDFs.

The source code of the application described herein as well as the trained model are publically available. Additionally, we created a web application accessible at *decimer.ai* to ensure that the segmentation algorithm becomes widely usable.

### Implementation

The DECIMER Segmentation backend mechanism was built using Python 3 with Tensorflow 2.3.0 [10]. It mainly consists of the recognition of chemical structure diagrams using a deep learning model and the subsequent expansion of the resulting masks. The web application is developed in Python 3 using the Django version 3.1.3 framework and React.js for the front-end. The implementation details of the key elements as well as the complete workflow which accepts a pdf document as an input and returns the segmented chemical structure diagrams as an output are described below.

### Deep learning algorithm

For the chemical structure detection, a model utilizing the Mask R-CNN network [11] was trained where the Mask R-CNN implementation published by the Matterport team [12] was used with some modifications to work on Tensorflow 2.3.0 with Keras at the backend.

The dataset used for training the model is based on 994 articles from the *Journal of Natural Products* which were chosen arbitrarily from all available issues. We converted the pages of these articles into JPEG images (96 dpi) using the Python pdf2image package [13] and deleted all images that did not contain any chemical structure diagram. After deleting pages which did not contain any chemical structure diagrams, there were a total of 1820 pages. The VGG image annotator tool [14] was used to manually annotate the chemical structure diagrams present in each image. Each depiction of a chemical structure was annotated by defining a polygon around it. If there were mechanism arrows or numbers within the structure, these were also included. Other objects like reaction arrows or labels around the chemical structures were not included. This resulted in 9992 annotated regions in the images which each contained one structure diagram (approximately 5.5 annotated structures per image). This dataset was split randomly into a training and validation subset of 90 and 10% respectively.

The model used the hyperparameters pre-defined by the Matterport team, furthermore, we used a batch size of two images per batch, learning rate of 0.001, learning momentum of 0.9, 500 steps per epoch and 50 steps for validation. The model was trained on a compute-server equipped with an Nvidia 1080Ti GPU, 64 GB of RAM and two Intel(R) Xeon(R) Silver 4114 CPUs. The training started from the pre-trained COCO weights provided by the Matterport team. The layers that could not be imported from the pre-trained weights of the model due to different amounts of classes (network heads) were trained for an initial 100 epochs, then the complete model was fine-tuned for another 100 epochs. During the whole training process, the parameters remained the same. This took approximately 26 h in total.

When applying the resulting model to an image of an article page, it returns masks which indicate whether or not a pixel in the original image belongs to a chemical structure diagram. These masks are binary matrices with the first two dimensions of the input image which can contain the values *True* or *False*. This means that every pixel in the original image has a corresponding value in the mask that defines whether or not this pixel is part of a chemical structure depiction. The positional information given in the masks can then be used for the segmentation of the chemical structures.

### Mask expansion algorithm

**Fig. 2:**
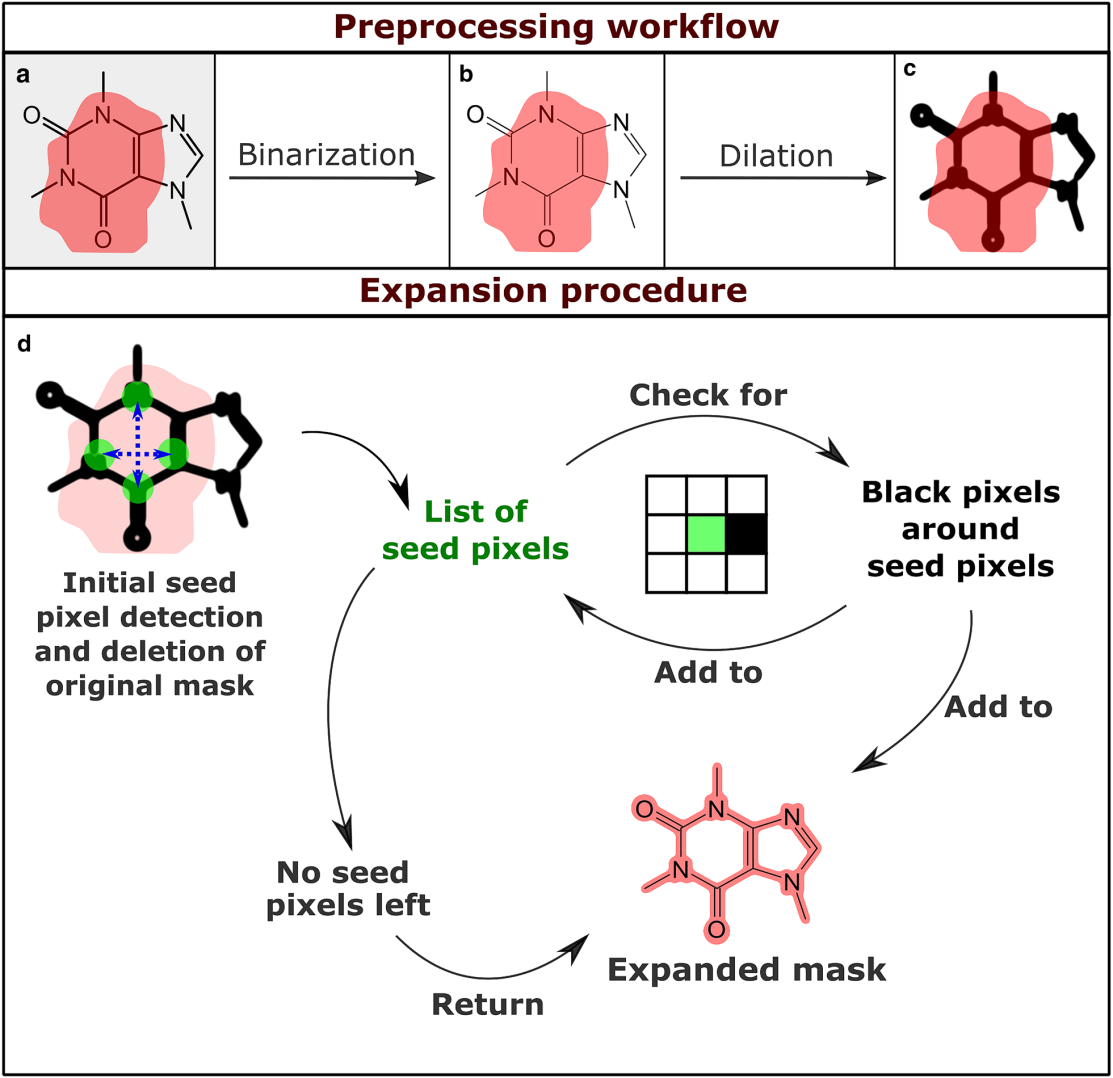
Mask expansion workflow: During the preprocessing workflow, the original image (**a**) is binarized. The overlaying red patch represents an incomplete mask which is returned by the model. The resulting binary image (**b**) is then dilated to fill gaps within the structure (**c**). This is followed by the expansion procedure (**d**) where the mask is reconstructed by tracing the connected set of non-white pixels starting from a list of seed pixels until no further connected non-white pixels can be found in any direction. This ensures the segmentation of complete chemical structure depictions

A common problem with the masks generated by the Mask R-CNN model is an unwanted partial coverage of chemical structures only: The model did correctly recognize the chemical structure diagrams on a given page but did not cover them completely (Fig. 2, top row). Therefore, a custom mask expansion algorithm was developed which takes an image and a mask and creates a mask that covers the previously partially detected objects completely.

The expansion workflow begins with the binarization of the input image using a high threshold as recommended by the developers of CSR [6]. The binarization ensures that a non-white background or relicts from low-quality scans are filtered. Then, a binary dilation is applied to turn chemical structure depictions into connected objects, closing, for example, the gaps between an element symbol and its adjacent bonds with non-white pixels. The kernel object used for the dilation is a square with a resolution-dependent size.

Then, the initial seeds for the expansion are determined. For this, the center of the mask is defined as the position in the middle between the highest and the lowest x- and y- coordinates of *True* values of the mask. If the resulting center point is not covered by the mask due to its asymmetric shape, the center point is defined as a random point between the highest and lowest x-coordinates which is covered by the mask. Based on the center point position, the algorithm attempts to determine four black pixels which are covered by the mask in four different directions. If at least one seed pixel is found, the original mask is replaced by a matrix of the same shape which only contains zeros and the expansion is initiated. If no seed pixels have been determined, objects on the contours of the mask are detected as seed pixels. In this case, the original mask is kept and only expanded based on the seed pixels.

The resulting list of seed pixel coordinates is used in the expansion procedure. The eight surrounding pixels of every seed pixel are examined. If one of them is black and not already covered by the mask, the mask is edited to cover it and it is added to the list of seeds. This recursive procedure leads to the inclusion of a complete object in the mask even if the original mask had not covered it completely. This outlined procedure is illustrated in Fig. 2.

### The complete tool

**Fig. 3:**
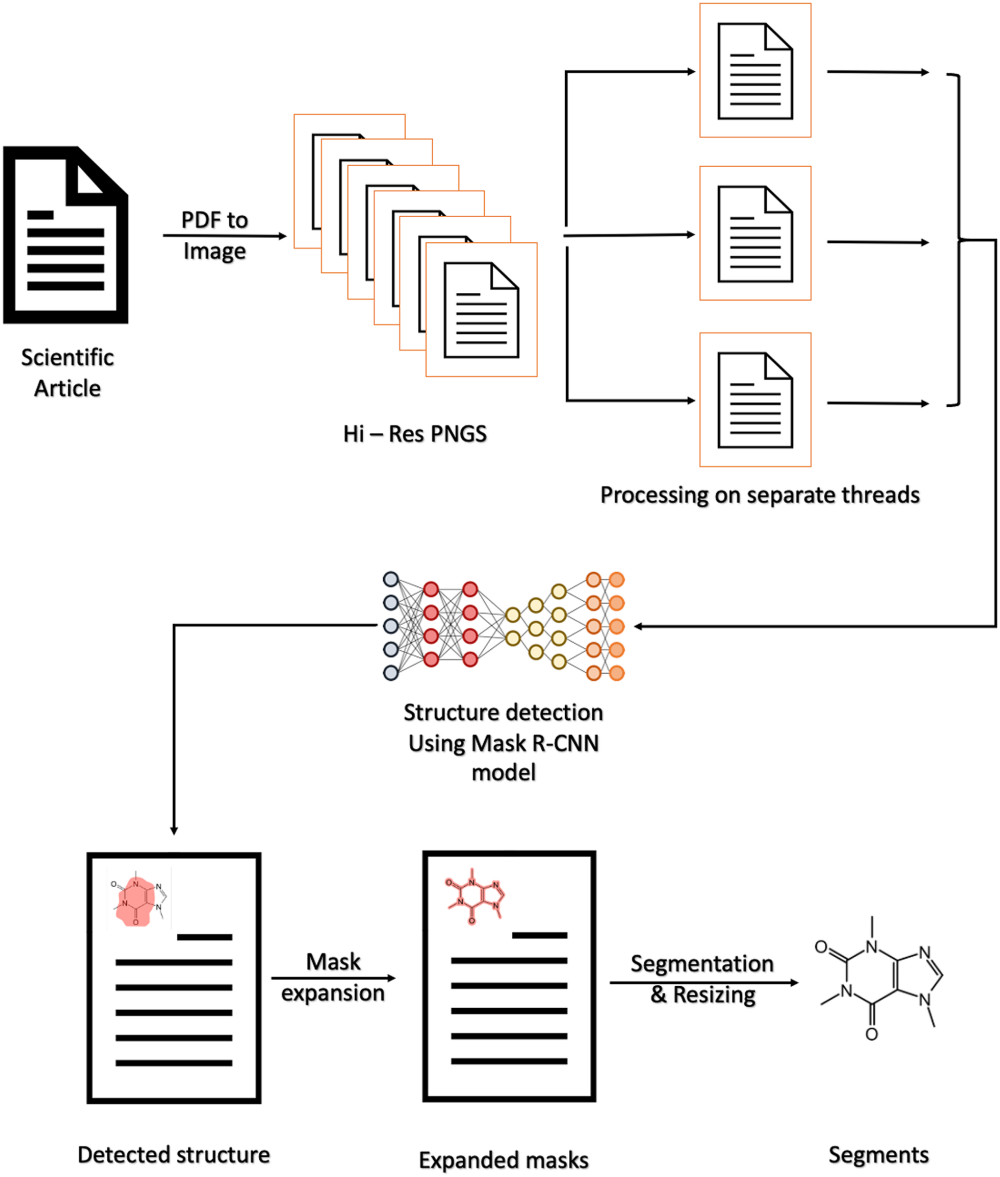
Visual representation of the complete architecture of the tool

DECIMER-Segmentation accepts PDF documents as input and returns grayscale images which contain the segmented chemical structure diagrams. Figure 3 illustrates the workflow.

All pages of the given input PDF document are converted to separate PNG images. All the images are stored in a folder with the name of the input PDF file. During the following procedure, the processing of each image can be parallelized. The structure detection model is initialized for each thread and generates the masks which define the positions of the chemical structure diagrams in the given image. Subsequently, these masks are processed by the expansion algorithm.

The final masks and images are then processed in a segmentation procedure. First, each segment is converted into a grayscale image. Then the maximal width and height of every mask are determined. With this information, an empty image with the dimensions of the resulting segment is created and the chemical structure diagram is placed in it. After all the segments are generated, they are resized into separate square images. These segments are displayed to the user at the end in the web application or saved locally.

### Decimer.ai web application

The single-page web application (SPA) is freely available at https://decimer.ai and allows DECIMER usage without any local installation. It is implemented with the Django framework version 3.1.3 to manage the back-end and the API and with the JavaScript React.js library for the front-end. The SPA allows the user to upload a PDF file of a research article, performs image segmentation on it, and returns the extracted molecular images. The latter can be downloaded. The user can also click on the “I’m Feeling Lucky” button, to randomly select a recent article from the Open Access journal *MDPI Molecules* and run the segmentation on it.

## Validation

### Methods

In order to evaluate the performance of DECIMER Segmentation, we processed 25 articles from the *Journal of Natural Products*, 25 articles from *Phytochemistry* and 25 articles from *Molecules*. None of these journal articles were included in the training dataset. The 75 articles contained a total of 777 pages (365 in *Molecules*, 228 in *Phytochemistry,* 184 in *Journal of Natural Products*) and contained 887 segmented images (398 in *Molecules* 183 in *Phytochemistry*, 306 in *Journal of Natural Products*). We then manually inspected all segmented images to determine if they contain a complete chemical structure diagram or additional objects such as labels or reaction arrows. Furthermore, we determined the number of additional missed structure diagrams on the pages where structures had been determined.

## Results and discussion

Without the application of the mask expansion, 81.6% of the segmented images contained complete chemical structure depictions (80.7% in *Molecules*, 83.5% in *Phytochemistry,* 81.7% in *Journal of Natural Products*). Here, a *complete detection* means that the structure was completely covered by the mask. It is necessary to mention that 9.4% (11.1% in *Molecules*, 5.5% in *Phytochemistry,* 9.4% in *Journal of Natural Products*) of these segments contained additional surrounding objects like labels or reaction arrows. Mechanism arrows or atom numbering were not counted as additional objects here as they are often positioned within the structure itself.

**Fig. 4:**
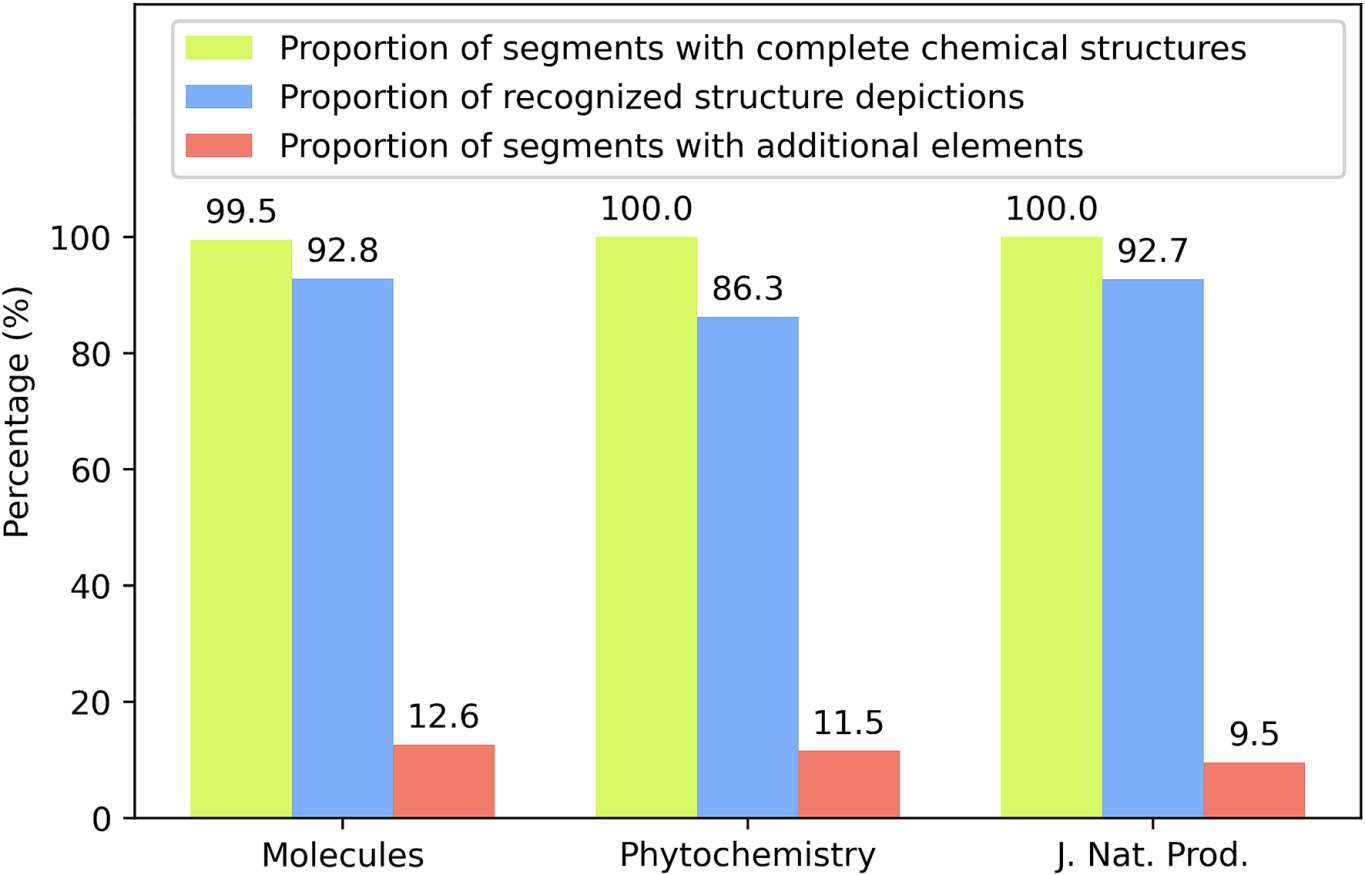
Overview of the validation results of DECIMER Segmentation

When the mask expansion was added to the procedure, the proportion of completely segmented structures increased to 99.8% (99.5% in *Molecules*, 100% in *Phytochemistry,* 100% in *Journal of Natural Products*). Among the validation results there were only two segments which did not contain a chemical structure diagram at all. Unfortunately, the proportion of segments that also contained additional objects rose to 11.2% (12.6% in *Molecules*, 11.5% in *Phytochemistry,* 9.5% in *Journal of Natural Products*). On average, 91.3% of the chemical structures were detected by the model (92.8% in *Molecules*, 86.3% in *Phytochemistry,* 92.7% in *Journal of Natural Products*). These results which represent the final output of DECIMER Segmentation are illustrated in Fig. 4.

Throughout the data used for validation, 885 of the 887 segments contained a complete chemical structure depiction. Given the fact that the model was only trained on articles from *Journal of Natural Products*, it is interesting to note that DECIMER Segmentation performs comparably well on the subsets of *Molecules* and *Phytochemistry* articles. This elucidates the general applicability of DECIMER Segmentation—it is capable of detecting chemical structures in the printed scientific literature in general, independent of specific publisher formats.

**Fig. 5:**
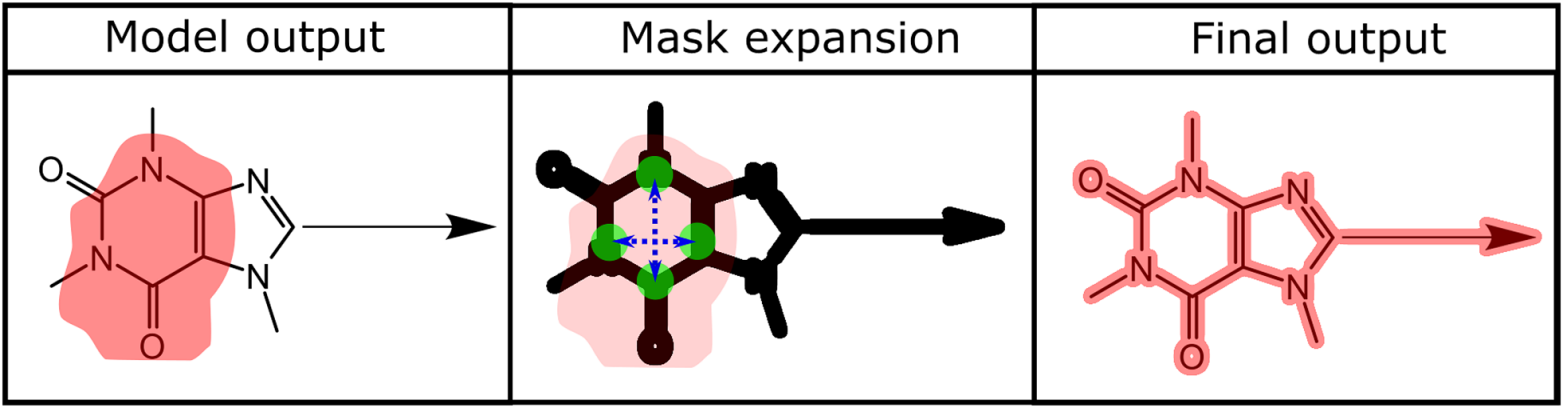
Exemplary illustration of the wrong inclusion of a reaction arrow in the mask output

The inclusion of additional objects in approximately 11% of the segments is, in many cases, caused by surrounding labels or arrows, which were placed closely to the actual chemical structure diagram by the human creator of the graphic (see Fig. 5). It is worth mentioning that the mask expansion sometimes aggravates the problem. For example, in some cases, the tip of a reaction arrow is covered by the mask which is returned by the model. If the arrow is close enough to the structure, the mask expansion leads to its complete inclusion. In other cases, an initially included reaction arrow may be excluded after the mask expansion if it is not too close to the structure. This is the advantage of choosing seed pixels in the center of the mask which is returned by the model. In an earlier version of DECIMER Segmentation, every pixel of the mask in the model output was included in the final output and the seeds for the expansion were set on the contours of the original mask. This led to wrongly included objects in above 20% of the segments because all objects close to the structure *and* all objects that were wrongly included in the model output were included in the final output. Hence, the mask expansion from seeds in the mask center led to significantly improved results.

During the mask expansion, the application of a binary dilation is necessary to turn the chemical structure depictions into connected objects. This can lead to nearby objects being connected with the structures. This could be addressed by using a smaller kernel for the dilation. The dilemma is that a smaller kernel leads to more cases where the structure is not one connected object which leads to incomplete expansion results. Hence, reducing the amount of wrongly included surrounding objects would necessarily lead to a reduction of the complete segments.

When processing pages parallelly on four threads, on average, the tool took about 1.89 min to process a single article with an average amount of 10.4 pages per article. The time required for processing depends on the number of pages and the number of chemical structures on each page. The numbers mentioned above correspond to an average processing time of 10.9 s per page.

## Conclusion

The DECIMER Segmentation tool and the web implementation on decimer.ai for chemical image segmentation are a complete open-source implementation for the segmentation of chemical structure depictions from the published scientific literature.

With the help of deep learning, our method is capable of distinguishing between chemical structures and other content on a page. By applying the system to images, we can mine information from scanned documents which are not available in markup file formats. This allows us to extract information even from the old articles which are only available as scanned files. With the implemented mask expansion process, we are able to segment chemical structure diagrams from the publications completely in high quality.

Although the model was only trained on articles from the *Journal of Natural Products*, we were able to see that the application works well on publications from three different publishers. In future, the detection accuracy of the model can be improved further by training it on an increased amount of publications. In its current state, DECIMER Segmentation can reduce the workload for those who are responsible for the manual creation and curation of chemical databases immensely and could eventually contribute to the full automation of this task.

## Data Availability

The DECIMER—Segmentation web app can be accessed at https://decimer.ai The code for DECIMER—Segmentation is available at https://github.com/Kohulan/DECIMER-Image-Segmentation. Project name: DECIMER. Project home page: https://decimer.ai. Operating system(s): Linux, MacOS and Windows 10. Programming language: Python 3. Other requirements: Python packages: Tensorflow 2.0 or above, pillow, opencv-python, matplotlib, scikit-image, imantics, IPython and pdf2image. License: MIT. Any restrictions to use by non-academics: Not applicable.

## Abbreviations

API: Application Programming Interface
CNN: Convolutional Neural Network
CPU: Central Processing Unit
CSR: ChemSchematicResolver
DECIMER: Deep lEarning for Chemical IMagE Recognition
GB: Gigabyte
GPU: Graphics Processing Unit
JSON: JavaScript Object Notation
JPEG: Joint Photographic Experts Group
MDPI: Multidisciplinary Digital Publishing Institute
OCSR: Optical Chemical Structure Recognition
OSRA: Optical Structure Recognition Application
PDF: Portable Document Format
PNG: Portable Network Graphics
RAM: Random Access Memory
R-CNN: Region-Based Convolutional Neural Networks
VGG: Visual Geometry Group
